# Analysis of the Correlation between Nutritional Status and Quality of Life of Patients with Gynaecological Ovarian Cancer during Postoperative Chemotherapy

**DOI:** 10.1155/2022/9877354

**Published:** 2022-06-22

**Authors:** Xiaoming Shi, Yuchun Lv, Peiqi Wang, Xin Yang, Shengjun You

**Affiliations:** Department of Obstetrics and Gynecology, Quanzhou First Hospital Affiliated to Fujian Medical University, Quanzhou, Fujian, China

## Abstract

Ovarian cancer mortality is on the rise in China. Surgery followed by adjuvant chemotherapy is the most extensively used treatment for tumour recovery. An excellent nutritional condition prior and throughout treatment serves to improve the quality of life and, as a result, the treatment result. The goal of this research was how diet affected the functioning standard of those living in carcinoma who were receiving postoperative treatment. BMI was utilised to evaluate nutrition, accompanied by albuminemia, prealbuminemia, and serum C-reactive protein, that is used to evaluate excessive catabolism. The QLQ-C30 questionnaire assessed standard of living. The performance status of the patient is decided with the help of the WHO performance scale for cancer patients. The study identified the statistically significant relationship between the performance status and hypercatabolism in the global health (quality of life) of the patient. While body mass index is often considered as a standard for assessment of nutritional status, it has affected only the cognitive function of the patient. In this study, we have concluded that in addition to direct measurement of the BMI, other clinical parameters such as serum CRP should be considered to get a better outcome of chemotherapy.

## 1. Introduction

Epithelial ovarian cancer is the common kind of gynaecological cancer in women in the developed countries, and it is also the greatest cause of disease mortality in women [1]. In the US, the prevalence of melanoma across all females have declined dramatically (−1.03 percent), among females diagnosed at the age of sixty-five (−1.09 percent) and among females diagnosed at the age of sixty-five and older (−1.09 percent), according to the National Cancer Institute (−0.95 percent). All females had an important quantitatively average yearly variance in survival at 12, 24, and 60 months (0.19 percent), and the estimated yearly percentage change at 60 months (0.72 percent). Severe form (III or IV) of the illness, on the other hand, had a poor prognosis by the end of the 5th year, with even less than 50 percent for women aged sixty-five years and much less than 30 percent for women aged sixty-one years. The data demonstrated a considerable variance in the occurrence of ovarian cancer rates around the world; the lowest levels are in China, while the largest percentage is in the Russian Federation and the UK [2].

**Figure 1:**
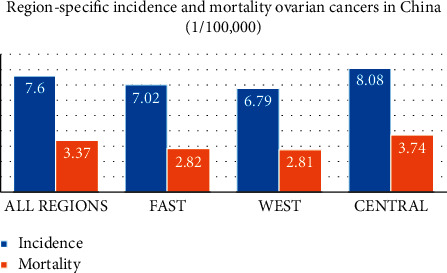
Regional occurrences and rate of mortality due to ovarian cancer in China.

Figure 1 emphasises the regional occurrences and rate of mortality due to ovarian cancer in China. Ovarian cancer occurrence and death rates in China are minimum compared with the global average, whereas the people belonging to the northern part of America, western part of Europe, and eastern part of Asia are preferably higher compared with the south-eastern part of Asia. Ovarian cancer mortality is on the rise in China, particularly in the areas of Inner Mongolia, Jilin, Tianjin, and Hebei [3].

Because of a lack of adequate routine screening, approximately 25% of cases of ovarian cancer are discovered at a preliminary phase before the cancer has spread. In the vast majority of these situations, surgery can completely cure the condition, and the five-year overall survival for early-stage ovarian cancer is quite well over 90%in these instances. The question of whether or not postoperative chemotherapy should indeed be employed in the identification of acute ovarian cancer is currently being debated. To conclude, many individuals think that chemotherapy can indeed be ignored in these instances at the very least and that individuals should be urged to participate in rehabilitative and investigative follow-ups. This belief is supported by research. Preoperative medication preceded by duration debulking surgical intervention was found to be no more efficacious than prevailing debulking multiple surgeries symptomatic as a conceivable therapy for advanced with thick and heavy ovarian cancer in a survey performed to select the optimal remedy for ovarian cancer. This procedure may be used as a treatment modality or as a follow-up to medication [4–6] and has the goal of completely removing all visible disease.

## 2. Related Works

In [7], the author recognized the epithelial cancer level in conjunction with a platinum-based chemotherapeutic agent along with taxane. Treatment with chemotherapy should be considered when a patient has a severe illness or a tumour that has a low differentiation potential. In a hospital situation when optimal staging is not routinely performed or achieved, it may be necessary to take a more pragmatic approach.

The ability to withhold adjuvant treatment from patients who are already in the encapsulating stage of Internal audit grade 1 serous or endometriosis carcinoma has been demonstrated [8], and this treatment can still be provided to everyone who is unwell in the early stages of the disease.

People came from throughout China to participate in an interbridge study in 29 major hospital emergency departments in 14 Chinese cities. The contestants came from throughout the United States. They were evaluated for their nutrient intake and performance status (nutrition risk index and performance status). The researchers included 1138 cancer patients who were hospitalised as participants in their study. Malnutrition was observed in 41.3 percent of the patients in this study, according to the findings. As reported by the National Research Institute, dietary deficiencies affect 51.4 percent of the population.

Preferred score (PS) equalled zero in 50.3 percent of cases, 15.4 percent in 15, 13.9 percent in 14, and 20.4 percent in 3. The researchers found that compared with patients with a PS of 0–1, those with a PS of 3–4 had a 1.275 (95 percent confidence interval (CI) 0.250–0.488, *p*0.0001) higher relative risk of malnutrition [9].

Sleeplessness, constipation, financial issues, and menopausal symptoms were among the most often mentioned symptoms of menopause. It was discovered that higher educational levels, occupations, residence regions, family income, and treatment techniques were all statistically associated with overall quality of life and social functioning. Similar findings were obtained regarding the relationship between role functioning and cancer stage, treatment mode, and time since diagnosis. Cervical cancer patients' poor economic situation, along with their rural living surroundings, has a major detrimental influence on their total standard of living according to the WHO. When it comes to their sexuality, younger, better educated patients are more concerned than those who are older and less well-educated patients. Individuals who underwent a large number of therapies had larger problems with their quality-of-life assessments when compared with patients who just received surgery [10].

Following the diagnosis of breast cancer, research of 156 participants discovered that optimistic thinking, symptom distress reduction, social support, illness evaluation, consider giving coping mechanism, and overall happiness were all connected with a higher quality of life. Positive attitudes, social support, symptom distress, lymphoid nodular status, disease evaluation, and the employment of a give-in coping style were found to be significant predictors of quality of life, accounting for 66.6 percent of the total variation. Because of these results, researchers concluded that it is critical to assist females in reducing their symptoms of distress, positively appraising their illness, employing fewer negative coping mechanisms, remaining optimistic, and maintaining strong support system because most of these concerns have an implicit or explicit impact on quality of life [11].

It was decided to employ the EORTC QLQ-C30, an open-to-interpretation questionnaire that queried patients regarding changes in physical, psychological, and social attributes. The use of global HRQOL to assess overall quality of life was also decided upon by the group. Several of the patients were suffering from severe disease as a result of treatment for breast cancer that had spread to the bones or small-cell lung cancer that had spread to the bones. A quantitatively significant change inside the QLQ-C30 was evaluated by the result found on the variability of the scores on the questionnaire. A difference of 5 to 10 points on a scale from 1 to 100 was rated moderate on such a scale from 1 through 100. Minor changes were deemed to have occurred in patients with a score discrepancy between 10 and 20 points, whereas significant changes were considered to have occurred in patients with a score difference between 20 and 30 points. The level of change considered clinically relevant varies greatly from one demographic to another, including from one individual to another, when utilising anchoring [12].

When researchers looked at how pain and other side effects affected people's moods and sleep, they found that they were also linked to how much they ate, how tired they were, and how supportive their families were. Social support and knowledge of the disease were not linked to treatment preferences or preferences for how to deal with the disease, though. Pain goes hand in hand with quality of life. The organisation can come up with a complete plan for improving rehabilitation services for this group of people [13].

Several research findings in China have found that having a lot of money and having a lot of support from friends and family improve the quality of life of Chinese women who have already been diagnosed with ovarian cancer. People who have breast cancer should get a lot of help with both their money and their social lives, if such findings are true. Traditional Chinese medicine can help breast cancer patients improve their quality of life (QOL), but more research needs to be done to find out for sure. It is appropriate to carry the cure to Western societies in which it is not available or restricted if it has been proven to work [14].

In this study, researchers looked at and compiled the predictive ability of nutritional status in people with cancer, including their body mass index (BMI) and weight loss. They also looked at nutrition detection and treatment and biochemical markers. In both univariate and multivariate studies, the predictive nutritional index was indeed a significant predictor of patient survival, as shown in the data that were accessible to the public (PNI). It is said in the assessment that nutrition plays an important role in how cancer patients get sick and how long they live in this way. In more than 85% of patients with gynaecologic malignancies, doctors have found that they are not getting enough food. This can happen at the time of the diagnostic test or during treatment. In addition, 40% of the patient populations were very undernourished and needed help right away [15]. A study found that people with sophisticated stages of cancer were more highly probable to be malnourished than people with less advanced stages of cancer. Throughout the first month, the patient ended up losing 5% of his body weight. Having hypoalbuminemia of less than 2 g/dL is a sign of severe malnutrition because it means there is not enough food in the body. Ovarian cancer patients' standard of living is harmed by a number of things, including physical problems caused by rehabilitation side effects [16]. During a condition and treatment, the physician must also keep an eye on the patient's quality of life (QOL). They must use methods and therapeutic agents that take into account the patient's preferences and QOL but also avoid and cure essential symptoms as quickly as possible to keep the patient healthy [17].

Statistically, the link between quality of life and nutritional and effectiveness status was strong in the group that had the highest quality-of-life score (*p*0.05). As a result, this study found that people with poor quality of life (QOL) were more likely than others to have it. This was strongly dependent on the nutritious category and level of performance that the study looked at. Also, it was found that quality of life is a very good predictor of how well a person will do after they have cancer [18].

In a study of 97 people who had cancer, it was found that 66 of them were girls and 31 were men (see figure below). The ECOG was rated 0.88 out of 3 on a scale of 1 to 3. This means that the ECOG did well. People in the study were an average of 54.551114 years old and had a performance status of 0.88. As per the Subjective Global Assessment (SGA), 58 patients were found to be getting enough food, while 39 patients (40.21 percent) were found to be undernourished. The results of an ANOVA test show that people with the disease in the subjective global assessment group had better overall standard of living than those from the control group. It is important to run high-quality, long-term studies to find out how nutrition and its predictors affect the quality of life of people with ovarian cancer in China, and how that affects the effectiveness of chemotherapy [19]. In [20], the authors intended and found how well surgeons' assessments of residual disease (RD) and pretreatment computed tomography (CT) results matched up in women who had advanced stage ovarian cancer and had optimum surgical cytoreduction.

In [21], the authors found the overall survival (OS) and progression-free survival (PFS) of subjects with elevated and sophisticated epithelial ovarian cancer (EOC) managed until at minimum 60 months at a solitary gynaecological oncologist hospital. This research evaluates the rationalisation for medicinal alternative and the involvement of arthroscopic subassembly assessment in comparison with primary debulking surgery (PDS) to neoadjuvant chemotherapy as well as interval debulking surgery (NACT + IDS), categorising the information by tumor recurrence. The abdominal spreading of ovarian cancer makes it an excellent option for hyperthermic intraperitoneal therapy (HIPEC). Intravenous infusion therapy exposed the cancer to a substantially larger toxic concentration level than effectiveness of health care, and in vitro experiments have shown mixing hypothermia and platinum has a progressive cytotoxicity effect. Preclinical studies have shown that toxic medications are abundant in the peritoneum, with limited exposure and mortality [22].

In [23], the authors assessed the efficacy and safety of carboplatin/paclitaxel neoadjuvant chemotherapy followed by interval debulking surgery (NACT-IDS) for initial stage and PDS plus postoperative chemotherapy for advanced ovarian cancer. In [24], the authors suggested that the functional somatic syndrome (FSS) strategies for maintaining fertility, such as not removing the uterine body and keeping at least one ovary.

In [25], conservative treatment for endometrial cancer (E.C.Co.) is suggested. The Gynecologic Cancer InterGroup has authorised a global effort focused at recording individuals with endometrial cancer (EC) who have been treated conservatively. This study reports on the diagnosis and treatment and fertility results of intramucous, G2 endometrioid EC individuals. The ovarian cancer study reviewed focused on pharmaceuticals and immunological circuit blockers, postoperative radiotherapy or multiple simultaneous chemotherapy, and radiation therapy after laparoscopic procedure in precancerous cervical cancer, severe operation in precancerous cervical cancer, and prophylaxis and monitoring. [26].

In [27], the authors suggested that the ovarian, fallopian, and primary peritoneal cancers are usually diagnosed after the illness has progressed. Neoadjuvant chemotherapy accompanied with interval debulking surgery accompanied with adjuvant chemotherapy and primary debulking surgery accompanied with adjuvant chemotherapy are two methods of the standard therapy of these malignant tumours. In this paper, the Malnutrition Quality Improvement Initiative (MQii) and other malnutrition quality improvement plans (QIPs) have been found to be beneficial in detecting and treating malnutrition. Nutrition- or malnutrition-focused QIPs in cancer treatment, on the other hand, are little understood or documented. This study describes the limitations and possibilities for QIPs in the nutrition support of cancer patients and offers information to encourage translational research on quality improvement [28]. The European Society for Clinical Nutrition and Metabolism (ESPEN) has advised that only cancer sufferers get additional adequate nutrition; yet, however, little known about more optimal dietary treatment. The purpose of this research was to assess the evidence available for nutritional support in terminal cancer sufferers. [29].

Malnutrition is common in elderly cancer patients, with prevalence rates ranging from 25% to 85%. The ageing process is linked to a number of physiological changes that may have an impact on one's nutritional health. Malnutrition status in elderly cancer patients may be detected using screening techniques [30].

## 3. Methodology

This research was done as a prospective observational study at Quanzhou First Hospital affiliated to Fujian Medical University. Patients who underwent surgery for complete resection of epithelial ovarian cancer and planned for a platinum (cisplatin or carboplatin)-based and taxane (docetaxel and paclitaxel)-based chemotherapy were included for the study participation. The data were collected at baseline and before every cycle of chemotherapy.

### 3.1. Data Collection

#### 3.1.1. Baseline Assessment

When the patient is planned for the chemotherapy, the baseline assessment of the patient is done. The demographic data of the patient are collected from hospital records, including age, marital status, weight (in kg), and body mass index. The body mass index is calculated using the following formula:

(1) $$\begin{array}{c} \text{BMI}=\frac{\text{Wt}\left(\text{kgs}\right)}{{\left[\text{HT}\left(\text{m}\right)\right]}^{2}}. \end{array}$$

Before every cycle, general performance assessment of the patient is performed

Before cycle 1 of chemotherapy, the following assessments are performed:

  (i) Performance status is assessed using the WHO performance scale.  Table 1 depicts the performance scale of the World Health Organization (WHO), which holds the following fundamentals:
  (ii) Assessment of nutritional status is done by using weight (in kg)
  (iii) Body mass index calculated as per the above formula
  (iv) Albuminemia (less than 30 g/L), a late sign for malnutrition, is used in biological definitions
  (v) Biological definitions based on prealbuminemia (less than 110 mg/L), which is an early marker for malnutrition
  (vi) C-reactive protein (more than 15 mg/L) is used to define hypercatabolism [31]
  (vii) The need for enteral or parenteral nutrition was assessed by an independent physician as per the standards by nutritional recommendations of the European Society for Parenteral and Enteral Nutrition [32]
  (viii) Quality of life was assessed by using QLQ-C30. The QLQ-C30, a thirty-item questionnaire with 5 functional measures, indicates a great quality of life [12].

Before cycle 2 of chemotherapy, the following assessments were performed:

- Performance status is assessed using the WHO performance scale
- Assessment of nutritional status is done by using weight (in kg)
- Body mass index calculated as per the above formula
- Albuminemia (less than 30 g/L), a late sign for malnutrition, is used in biological definitions
- Biological definitions based on prealbuminemia (less than 110 mg/L), which is an early marker for malnutrition
- C-reactive protein (more than 15 mg/L) is used to define hypercatabolism

Before cycle 3 of chemotherapy, the following assessments were performed:

- Performance status is assessed using the WHO performance scale
- Assessment of nutritional status is done by using weight (in kg)
- Body mass index as per the above formula
- Albuminemia (less than 30 g/L), a late sign for malnutrition, is used in biological definitions
- Biological definitions based on prealbuminemia (less than 110 mg/L), which is an early marker for malnutrition
- C-reactive protein (more than 15 mg/L) is used to define hypercatabolism
- The need for enteral or parenteral nutrition
- Quality of life was assessed by using QLQ-C30

The research was authorised by the Medical Ethical Review Committee of Quanzhou First Hospital affiliated to Fujian Medical University and conducted in line with the Declaration of Helsinki.

### 3.2. Statistical Analysis

The qualitative data from the whole group of patients are gathered. Means, standard deviations, medians, and interquartile range (IQR) are often used to show how long a variable is. In this example, percentages are used to show qualitative variables. Using a paired Student's *t*-test, we looked at how the five functional QOL scores in the sample changed over time. We looked at how the scores changed between cycles 1 and 3. Student's *t*-test and Pearson's correlation coefficients were used to check for age, marital status, and nutrition at the start of the first cycle of chemotherapy, as well as at the end of the third cycle. These tests were used to figure out QOL scores at the end of the cycle. Multiple tests were done to compare different things (Bonferroni). Several linear regressions were used in multivariate analysis to look for characteristics that might be linked to QOL at the third cycle (5 function scores). When we looked at the function, we looked at each dimension as a separate dependent variable. It was important to pick variables that had a certain *p*-value so that they could be used in the models.

## 4. Results

### 4.1. Sample Characteristics at Baseline

The baseline characteristics of the patients are given in Table 2. From June 2016 to September 2019, 56 patients were included. The median age was 59 years (interquartile range 49–69). In the BMI point of view, 10.2% of the patients were less than ideal nourishment status at inclusion. During cycle 2 and cycle 3, 48 and 45 patients were checked, respectively.

### 4.2. Nutritional Status

Table 3 depicts the nutritional status of the patients during chemotherapy. This shows that 9% is severe and 91% is mild among the total participants, which indicated global adequacy in the application of nutritional guidelines. At cycles 1, 2, and 3, the adequacy was 35, 35, and 27 percent, respectively, according to the rigorous criteria. It was 95, 99, and 94 percent for the less restrictive definitions for cycles 1, 2, and 3, respectively. Throughout the observations, the patients' nutritional status remained nearly constant as before to each cycle. The nutritional status was found to be poor throughout the second cycle of chemotherapy, although no statistical significance was found.

### 4.3. Quality of Life

**Figure 2:**
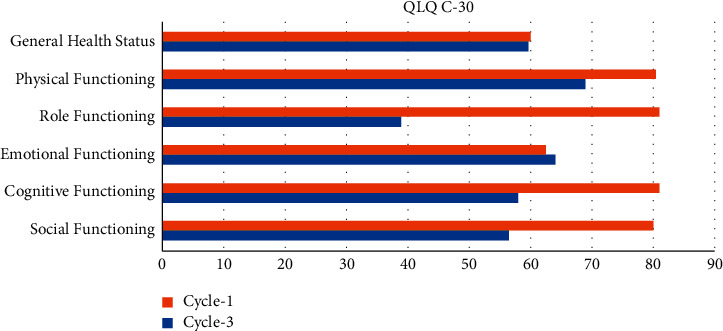
Quality of life at cycle 1 and cycle 3 of chemotherapy.

The functional scores of EORTC QLQ-C30 are shown in Figure 2. The cognitive function score was poor at cycle 3 compared with cycle 1.

Malnutrition may have a factor in deciding wellbeing. The results of the univariate analysis of relationships between QOL scores of functional measures (EORTC QLQ-C30) and socioeconomic and dietary condition at cycle 3 are shown in Table 4. A multiple regression was done to assess the link between QOL and nutritional status during the third cycle of treatment. Cognitive abilities were not affected by BMI.

Table 5 summarises the overall findings. It can be noticed that the performance status and CRP have a statistically significant effect on global health. The BMI has an impact on the cognitive function of the QOL assessed using the questionnaire.

## 5. Discussion

The primary emphasis of this study was on the nutritional state of patients undergoing adjuvant chemotherapy for ovarian cancer and its relationship to QOL. The baseline characteristics of 56 individuals were collected from June 2016 to September 2019. The median age of the participants was 59 years (interquartile range: 49–69). 10.2 percent of the patients had worse than optimum nutritional status at the time of inclusion, according to BMI. Despite the fact that various research projects have looked into the nutritional state of ovarian cancer patients, relatively few have looked into our group. In contrast to previous research, this study found that the patient group had adequate nutritional status. Only 2–19% of the recruited individuals were found to be malnourished. The ovarian cancer-related weight increase and the heterogeneous demographic of patients involved in the study could be the reasons. The reason for this is also due to the patients who have been chosen to be eligible for chemotherapy. Only healthy, well-nourished patients are considered suitable for chemotherapy, implying that the patients have a healthy BMI.

**Figure 3:**
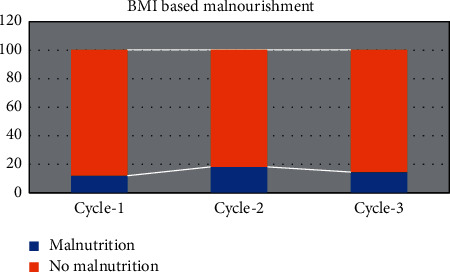
Analysis of BMI-based malnourishment in the patients during overall chemotherapy.

The nourishment status of the patients during the overall chemotherapy is depicted in Figure 3. It can be noted that the nutritional status remained practically unchanged throughout the cycle. Another factor could be that patients are generally well nourished prior to chemotherapy and are therefore well suited to the treatment. Prior to the second cycle, there is a reduction in nutritional status, as expected. Chemotherapy-related toxicity would manifest themselves here. Chemotherapy-related weight loss could be attributed to anorexia, nausea, and vomiting, all of which are postchemotherapy side effects. The knowledge gained during the second cycle has aided in maintaining the stringent nutritional protocol, preventing further deterioration in nutritional status during the third cycle.

The QLQ-C30 was employed for the assessment of QOL. Between the first and third cycles, the quality of life drops slightly. Chemotherapy appears to have a significant impact on cognitive function in particular. However, because there is no statistical significance, it is impossible to determine the impact.

Although age is thought to be a significant determinant in determining one's quality of life, the facts suggest otherwise. Furthermore, as previously documented, performance status, which indicates functional impairment, appears to be a sensitive determinant of the physical QOL component. Malnutrition can have an impact on an individual's social interactions, lowering their quality of life. Surprisingly, prealbuminemia was identified having a strong association with quality of life. Prealbuminemia reduced overall quality of life, but this is a new finding, and more research is needed before any conclusions can be drawn.

Only one literature concentrating on the specific role of CRP, a recognised measure of systemic inflammation and hypercatabolism, has been found to predict QOL to our knowledge [33].The following variables independently predicted the QOL of the patients according to a multivariate analysis; for example, anorexia medications in the pipeline include anamorelin, an oral ghrelin-receptor agonist with appetite-stimulating and anabolic properties [34], thereby people's quality of life should be improved.

### 5.1. Limitations

Before it is widely accepted, this study has a few drawbacks. The first is that we used BMI as a technique to assess malnutrition when, in an ideal scenario, weight loss would have been taken into account. Weight loss would be the proper approach to diagnose malnutrition in numerous well-developed nations, especially western countries, where the average BMI of their population appears to us to be obese, and hence weight reduction would be the right way to diagnose malnutrition. However, in the case of our country, this is not the case. ESPEN recently established a new diagnostic criterion that might be utilised to investigate inconsistencies in malnutrition assessment in this group of patients. Another flaw in this study is the sample's representativeness, which did not differ from earlier studies in terms of age or gender ratio. Despite the fact that the three most common cancers treated in France are digestive, genital, and haematological malignancies, head and neck carcinomas and sarcomas were common. This disparity is related to the different demographics that each of the participating centres manages. Furthermore, the minimal sample prohibits a more thorough analysis of relationships with QOL, particularly prior to inclusion, research into the nature of chemotherapy, chemotherapy combinations, and the concept of radiation and/or surgery. Finally, further studies that compare clinical guidelines in different nations (the United States and other European countries) might provide important information.

## 6. Conclusion

Through the results of the study, we could confirm that the nutritional status affects the quality of life, particularly the cognitive function during the course of chemotherapy, while all other functions of the patients remain unaffected during the chemotherapy. However, other factors including serum CRP should be assessed along the chemotherapy to improve the outcome of treatment. A strict adherence to nutritional recommendation could improve the overall nutritional status during chemotherapy.

## Figures and Tables

**Table 1:** WHO performance scale.

Grade	Explanation of activity
0	Fully functional, able to undertake all pre-disease activities with no limitations
1	Restricted in physically strenuous activities yet ambulatory and capable of light or sedentary employment, such as light housework or office work
2	Although the patient ambulates and does all self-care, she is not able to do any work duties. More than half of one's waking hours are spent up and about.
3	Only less self-care abilities, bound to bed or chair for about half of waking hours
4	Disabled completely. I'm unable to continue self-care. Confined to a bed or a chair.
5	Dead

**Table 2:** Baseline characteristics of the patients.

Characteristics		*N* (%) or mean ± SD
Age		58.4 ± 14.2
Marital status	Married	29 (69)
	Single	13 (31)
Performance status	0	25 (58.1)
	≥1	18 (41.9)
Baseline BMI		22.8 ± 3.1
	Malnutrition	5 (10.2)
	Normal	44 (89.8)

**Table 3:** Nutritional assessment.

Parameter	Category	Cycle 1 *N* (%) or mean ± SD *N* = 56	Cycle 2 *N* (%) or mean ± SD *N* = 48	Cycle 3 *N* (%) or mean ± SD *N* = 45
Clinical parameters				
Weight		58.7 ± 14.2	58.5 ± 13.9	58.6 ± 14
BMI		24 ± 4.2	23.8 ± 4	23.9 ± 3.9
	Malnutrition	7 (12.5)	9 (18.75)	7 (15.5)
	No malnutrition	49 (87.5)	39 (81.25)	38 (84.4)
Laboratory parameters				
Albuminemia		34.3 ± 4.5	34.6 ± 4.9	35 ± 5.4
	Malnutrition	6 (13.3)	6 (16.7)	6 (15.8)
	No malnutrition	39 (86.7)	30 (83.3)	32 (84.2)
Prealbuminemia		252.8 ± 71.8	253 ± 82.6	272 ± 170
	Malnutrition	1 (2.4)	2 (6.3)	1 (3.3)
	No malnutrition	40 (97.6)	30 (93.7)	29 (96.7)
Serum C-reactive protein	Median IQR	6.1 (2.0–17.2)	2.6 (1.1–16.7)	3 (1.0–12.4)

**Table 4:** Relationships between QOL scores of functioning scales (EORTC QLQ-C30) at cycle 3 and sociodemographic and nutritional status.

		Global health	Physical function	Role function	Emotional function	Cognitive function	Social function
		M ± SD/R	M ± SD/R	M ± SD/R	M ± SD/R	M ± SD/R	M ± SD/R
Baseline							
Age	*R*	−0.09	−0.07	−0.03	0.02	−0.05	−0.13
	*p*	0.38	0.60	0.77	0.95	0.70	0.28
Marital status	Couple	63.6 ± 24	75.4 ± 21.6	66.5 ± 29.9	72.9 ± 21.4	78 ± 26.9	77.2 ± 27
	Single	61.6 ± 23.5	71.2 ± 25.9	69.5 ± 31.1	78.4 ± 20	75.1 ± 26	72.3 ± 32.3
	*p*	0.70	0.44	0.68	0.29	0.66	0.51

T1							
Performance status	0	67.3 ± 22.3	79.43 ± 22	68.2 ± 32.5	76.0 ± 21.1	79.2 ± 25.2	76.4 ± 29.1
	≥1	57.4 ± 26.3	67.3 ± 23.1	67.1 ± 27.8	73.8 ± 20.6	74.1 ± 28.7	74.6 ± 27.8
	*p*	0.073	**0.024**	0.899	0.676	0.410	0.796
BMI	*R*	−0.15	−0.24	−0.22	−0.21	−0.091	−0.32
	*p*	0.2	**0.037**	0.61	0.08	0.45	**0.004**
Albuminemia	Malnutrition	58.2 ± 28.8	73.2 ± 21.9	70.1 ± 40.6	81.6 ± 16.7	81.6 ± 22.7	78.2 ± 31.6
	No malnutrition	64.2 ± 22.6	74.9 ± 22.8	68.3 ± 28.2	74.4 ± 20.9	77.7 ± 27.2	76.7 ± 25.6
	*p*	0.46	0.84	0.90	0.31	0.65	0.88
Prealbuminemia	Malnutrition	41.6 ± 35.5	70.1 ± 4.71	58.2 ± 58.8	58.4 ± 11.9	66.6 ± 47.2	99.8 ± 0.1
	No malnutrition	64.3 ± 23.9	75.3 ± 23.7	67.8 ± 30.5	74.7 ± 21.3	76.3 ± 27.4	75.8 ± 27.6
	*p*	0.21	0.33	0.68	0.280	0.619	**0.001**
CRP	*R*	−0.16	−0.17	−0.08	−0.02	−0.05	0.08
	*p*	0.23	0.18	0.57	0.90	0.61	0.56

T2							
Performance status	0	70.2 ± 24.6	83.4 ± 20.8	73.1 ± 33.9	76.1 ± 20.8	84.2 ± 23.8	82.9 ± 23.8
	≥1	61 ± 22.6	68.7 ± 24.1	67.6 ± 27.6	76.8 ± 21.1	73.4 ± 28.6	70.8 ± 31.1
	*p*	0.08	**0.007**	0.49	0.91	0.08	0.06
Weight change	*R*	−0.08	0.13	0.07	0.11	0.07	0.23
	*p*	0.43	0.28	0.60	0.39	0.65	**0.046**
BMI	R	−0.13	−0.22	−0.19	−0.20	−0.06	−0.26
	*p*	0.22	0.07	0.12	0.07	0.48	**0.017**
Albuminemia	Malnutrition	60.1 ± 25.9	73.2 ± 20.0	73.2 ± 30.5	84.1 ± 16.7	83.2 ± 23.7	83.5 ± 22.5
	No malnutrition	64.6 ± 24.1	74.6 ± 24.8	67.2 ± 29.1	74.3 ± 21.6	76.4 ± 28.5	75.6 ± 28.5
	*p*	0.61	0.90	0.55	0.18	0.48	0.41
Prealbuminemia	Malnutrition	38.7 ± 19.4	53.5 ± 13.5	33.5 ± 16.8	69.5 ± 21.1	66.8 ± 33.5	72.3 ± 34.5
	No malnutrition	64.8 ± 22	74.4 ± 24.5	69.7 ± 29.5	75.3 ± 22.3	76.7 ± 28.4	77.4 ± 28
	*p*	0.05	0.16	**0.03**	0.69	0.57	0.75
CRP	*R*	−0.33	−0.21	−0.17	−0.012	−0.0354	−0.04
	*p*	**0.015**	0.13	0.07	0.93	0.79	0.88

*Note*. Bold values indicate *p* < 0.05; BMI, body mass index; CRP C-reactive protein.

**Table 5:** Summary of the findings.

QOL factor	Assessment factor	*p*-value
Global health	Performance status	**0.011**
	Prealbuminemia	0.679
	CRP	**0.012**
Physical function	Performance status	0.061
	BMI class	0.4
Role function	Performance status	0.46
	Prealbuminemia	0.37
Emotional functions	BMI class	0.169
Cognitive function	BMI class	**0.02**
Social function	Weight change	0.207
	BMI class	0.9

## Data Availability

The analyzed data sets generated during the study are available from the corresponding author on reasonable request.
